# Monitoring and Detection Platform to Prevent Anomalous Situations in Home Care

**DOI:** 10.3390/s140609900

**Published:** 2014-06-05

**Authors:** Gabriel Villarrubia, Javier Bajo, Juan F. De Paz, Juan M. Corchado

**Affiliations:** 1 Departamento de Informática y Automática, Universidad de Salamanca, Plaza de la Merced s/n, 37008 Salamanca, Spain; E-Mails: gvg@usal.es (G.V.); corchado@usal.es (J.M.C.); 2 Departamento de Inteligencia Artificial, Universidad Politécnica de Madrid, Madrid 28660, Spain; E-Mail: jbajo@fi.upm.es

**Keywords:** multi-agent system, indoor location, WIFI, home care

## Abstract

Monitoring and tracking people at home usually requires high cost hardware installations, which implies they are not affordable in many situations. This study/paper proposes a monitoring and tracking system for people with medical problems. A virtual organization of agents based on the PANGEA platform, which allows the easy integration of different devices, was created for this study. In this case, a virtual organization was implemented to track and monitor patients carrying a Holter monitor. The system includes the hardware and software required to perform: ECG measurements, monitoring through accelerometers and WiFi networks. Furthermore, the use of interactive television can moderate interactivity with the user. The system makes it possible to merge the information and facilitates patient tracking efficiently with low cost.

## 1. Introduction

Nowadays telemedicine and home care are becoming increasingly important in society due to advances in sensor technology [1], devices, communication networks [2] and especially as the result of an aging population in Europe [3]. It is estimated that 50% of the population in Europe will be over 60 years old in 2040, while in the USA it is estimated that one in every six citizens will be over 65 years old in 2020 [3]. In addition, people over 85 years usually require continuous monitoring [3]. For this reason, it is necessary to create systems that can appropriately monitor the users [4] and that are easily adaptable to sensor networks and hardware from different manufacturers. The high price of the components to install at home is a key problem in home care. For this reason it is necessary to develop systems that are affordable. This paper proposes an architecture based on the PANGEA platform [5]; it allows embedding agents in different hardware to facilitate and perform careful monitoring of home users.

Context aware systems use wireless sensor networks to collect information from the environment and act according to the retrieved data [6]. They have evolved and been applied in several fields such as medicine [4]. One of the key features in context aware systems is adaptability, given that different users have different needs, as highlighted in [7]. For this reason the systems must be adaptable according to the needs of the users. Some studies make use of cloud computing or multiagent systems [4,8] to manage the available distributed sensors. Virtual organization of agents can be used to achieve this adaptability, as they allow for the inclusion of new roles and for system behavior to adapt dynamically [5,9]. The BISITE research group has developed the PANGEA [5] platform, which facilitates the development of agents in light devices and the integration of different hardware.

This study proposes a system based on virtual organizations of agents that allows patient monitoring and tracking by incorporating different hardware systems. The PANGEA platform was specifically used [5] as it allows the development of virtual organizations of agents and facilitates the deployment of agents in lightweight devices. The system incorporates different virtual organizations to monitor patients at home and it includes roles to locate and monitor users, to monitor cardiac function, and to allow the user to interact with the system through a television. The location system is based on probability to determine the user's location. It is based on Received Signal Strength Indicator (RSSI) signal levels received from different WiFi routers. The accelerometer of the mobile device is used to avoid continuous fluctuations in the location system due to changes in the signal levels; this makes it possible to detect the steps taken by the user and calculate his or her location through RSSI levels. This motion information will be of special interest with the electrocardiogram, since the user will not have to manually record physical activity. Electrocardiography (ECG) incorporates the necessary hardware to send the data to the system via Bluetooth. In addition, the system incorporates a basic analysis of the ECG for sending alerts. Finally, the user can interact with the system through the television by means of a Raspberry Pi, which allows the home to be continuously connected to the Internet. The paper is structured as follows: Section 2 discusses the state of the art; Section 3 presents the proposal; Section 4 details the case study; and finally Section 5 includes the results and conclusions.

## 2. State of the Art

Home care systems are becoming more prevalent in society due to an increase in the population of the elderly [3]. Home care [10], elderly care [3] or telemedicine systems [7] have thus become quite common. The functionality of home care, elderly care or telemedicine systems varies according to the type work. These systems also tend to merge with sensor networks to create context aware systems [4,10–12]. In [13] we can find one of the earliest references to context aware systems; it was a location system in offices. Subsequent applications have emerged in the medical field such as [14], which allows the exchange of information among hospital workers depending on a patient's status, availability of resources or devices. Later we can find references to context aware systems based on multi-agent systems, for example, the authors in [8] propose a set of agents that interact with the different sensors in a room, and other systems such as [15] can send alerts when upon detecting an anomalous situation. Other recent studies such as [16] develop a caregiver platform to manage and fuse information gathered from multiple sensors. While the applications are diverse, the study in [4] clearly indicates that location is an important aspect to consider in this kind of system. This study [7] reviewed different works in telemedicine, and highlights the relevance of creating adaptive systems that match user needs. This property is one of the characteristics of virtual agent organizations.

Location and identification systems based on sensor networks help locate and identify items in an environment [11]. In addition, the information provided may be used to identify and analyze user behavior, and detect abnormal situations. A proper tracking system requires different technologies, as indicated in [17]. The most common technologies are Radio Frequency IDentification (RFID) [3,18], ZigBee [19,20], WiFi [21,22], Bluetooth [23], Ultra-wideband [24], *etc.*, In addition to technology, there are different algorithms that can be applied such as trilateration [25], signpost, fingerprint [21]. The applied algorithm can achieve higher accuracy depending on the technology used. In this case, the required precision level was at the room level to locate a user in the area at any given time. Based on this characteristic, the technology and algorithm were selected. While ZigBee technology provides greater accuracy, its price is very high and was therefore discarded as an option, leaving active RFID and WiFi as the most viable alternatives. It also seems that Bluetooth is gaining ground once again with the appearance of ibeacon; however the 4.0 standard Bluetooth presents incompatibilities with many devices. Our research group has already developed several studies along these lines, specifically with RFID [3], WiFi [21], ZigBee [19], thus providing us ample experience in the management of these systems. As a result, we need only adapt this experience to the specific parameters of the problem. One of the problems with these systems is that RSSI signal levels vary considerably even though the user remains stationary; with the exclusive use of this system would appear to be in continuous motion even when they are sitting down. Additional information would be required to prevent this behavior; hence, accelerometers are included to create a pedometer to detect the motion of the users.

There are currently various commercial systems to detect and count steps, including FitBit, Jawbone [26], and Nike Fuelband [27], which are priced from 99€ to 139€. Given the fact that this price is approximately equivalent to the cost of a smartphone, it seems to be a high price to pay for the simple function it provides. In addition, the error rate is high for slow walkers, who are precisely the type of users that the systems target. The authors in [28] provide a rather complete analysis of the accuracy of FitBit with regard to speed. In addition to the imprecision in analyzing slow walkers, the system has also been shown to have other inaccuracies, such as detecting steps taken by the user when the user is driving. As a result, it would be preferable to have a mobile app do the work of an accelerometer at a much lower cost. These systems are based on the analysis of mobile accelerometers [27] and/or the state of the gyroscope [29], which they use to detect steps. There are several algorithms to detect steps; the most common involves establishing thresholds which, when they are exceeded, count as a step [27]. Other studies establish templates and compare the templates with the accelerometers to detect steps [30]. In the market, there are many applications that count steps, with the best known being, perhaps, the Runastic pedometer or Noom Walk. They work quite well but they may also recognize steps when the user is not walking. The accuracy of these systems is high when the user is walking slowly, as demonstrated in this work [26].

The information fusion of multiple sources may improve the results in indoor location systems [31]. The authors in [32] include information about indoor location systems using information fusion. We can also find studies that monitor daily activities according to the changes in the sensors of a mobile phone [33]. These studies usually use classifiers over the sensors to detect and classify activity; however, the number of different detected behaviors is limited. Information fusion is also applied in outdoor location systems; for example, in [34] the authors combine information gathered from GPS accelerometers and gyroscopes in order to detect trajectories. Similar, the present study could be adapted to an indoor location system using WiFi and combining the information from sensors to calculate the final position of the users. The use of cameras has been considered [35] although it would be necessary to introduce an additional algorithm to recognize people.

Currently, there are many studies on ECG that are very specialized in very different ways. Typically the majority are associated with the signal analysis of holters to detect P and T waves, and the QRS complex. Likewise, there are many studies to analyze the data of a holter and obtain the QRS complex, as shown in [2,36–39]. Some of these studies are designed to work in real time [36,40], which facilitates automatic processing at runtime—a key aspect in context aware systems. These studies are primarily based on the analysis of signals in time domain [36,40] or in the frequency domain [39], but most of them are based on the application of filters before extracting the information [36,40]. Signals are quite simple to analyze visually, but automatic processing is not always easy due to the noise and the change in the amplitude of the waves. In this regard, there are already numerous papers with many solutions. We have, therefore, simply reused some of the algorithms provided in literature and shown to have good results. The selected algorithms are fast and the results easy to interpret, as shown in both [36,40]. Once the P and T waves and the complex QRS are detected, an automatic diagnosis can be performed by using databases with different pathologies, the most common being MIT-BIH Arrhythmia, on which classifiers as SVM [41], KNN [42], decision rules [43] are applied. Given that any classifier can be applied to the database to generate a predictive model, this field was not considered very important. Some systems allow remote monitoring of users through web applications [44] and also provide further analysis on the data to detect pathologies, while others use mobile devices for real-time monitoring [45]. The main problem is that the hardware used is usually quite expensive. The cost of commercial holters such as BMS1200, or the DigiTrak XT Holter Recorder exceeds €20,000, making them far too expensive for widespread use; they also require low cost hardware to carry out these tasks. Moreover, a more effective analysis would require gathering information from the context to facilitate the doctor's work and provide details about the activities the user is engaged in.

## 3. Proposed System

**Figure 1.**
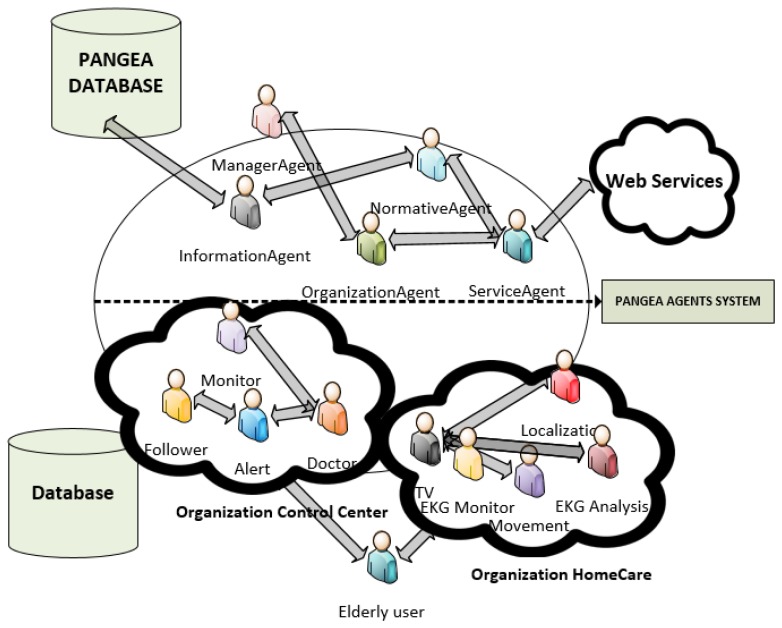
Virtual organization of the system.

The system proposed in this paper is based on a virtual organization of agents that monitors user information. The virtual organization of agents includes agents located in a control center and others located in the user's devices. Figure 1 shows the virtual organization of agents. The virtual organization has been created with the PANGEA platform [5]. More information about the architecture can be seen in [5]. The image shows the information associated with the system and includes only the specific platform agents, although they are not detailed in this study.

The first organization is the control center, which includes the following roles: tracking, monitor, alert, doctor. The tracking role is responsible for tracking the users to verify that the patient is following the daily routine. This role interacts with the agent with the role of television to interact with the user. The monitor role is responsible for retrieving status information from the sensors and sending alerts to the doctor or to the user through the television using the alert role. If necessary, the monitor role can store the information for the doctor to review at a future time. The alert role receives emergency notifications from the tracking or monitoring role and sends the information to the right agent with the appropriate role. The doctor role corresponds to the doctor or a person who is responsible for monitoring the condition of the patients. During a simple monitoring, this agent simply introduces information on the treatment of patients and enters reminders that are displayed through the user's television.

In addition, the system has home organization, which includes the following roles: television, location, movement, EKG analysis. The agents are deployed on the Raspberry Pi, on the mobile device, and on the Arduino. This is the reason for using the Pangea platform, since it is possible to include lightweight agents on low performance devices. The agent that implements the location and movement roles is deployed in the mobile device; these roles are discussed in Sections 3.1 and 3.2. The agent with the monitor role is in the Arduino device, while the agent role analysis is located in the Raspberry due to the limited processing power of the Arduino device. These roles are discussed in Section 3.3. Finally, the agent with the role of television is in the Raspberry, and is detailed in Section 3.4. There is an organization for each of the users.

### 3.1. Location System

The location role carries out the location process in a home care organization. The correct technology and location algorithm must be selected for a location system. Given the available technology, the best alternative for a low cost system is WiFi because the devices are cheap and it is possible, in many cases, to reuse the user's own devices. In addition to the technology it is also necessary to select the algorithm. In this case the alternatives were clear and they were totally dependent on the technology to be used. Technology such as trilateration is not feasible when there are many obstacles, and we want to limit the number beacons. Signpost would require installing many beacons to obtain good results. We chose to use fingerprint as the most suitable alternative to obtain adequate precision at a low cost, although it requires calibrating the environment.

**Figure 2.**
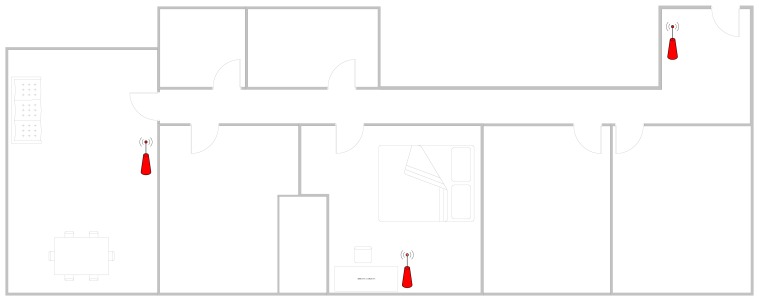
Distribution of the Access point in the flat.

The calibration process involves taking measurements at different locations of the person's home. These measurements will then be used to calculate the location of the user. Before taking the measurements, it is necessary to place at least three access points in different areas throughout the home to determine the exact location of the user. While it would be possible to use WiFi networks from neighbors, it is not recommended because the location of the access points can vary or even turn off. In the end, the distribution of access points created for each home is similar to the distribution shown in Figure 2.

**Figure 3.**
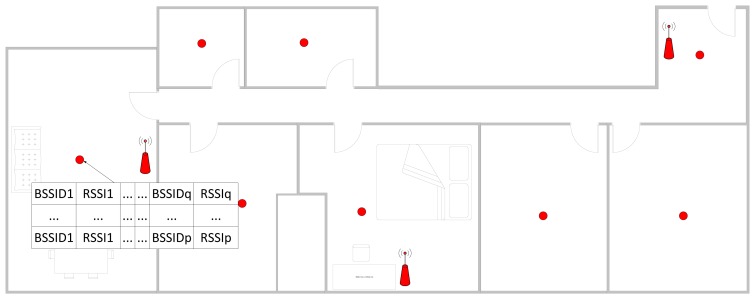
Calibration Process.

Using this distribution, we proceeded to measure the RSSI levels in different locations. It is not necessary to know the current location of the access points and it is not necessary to enter the information of the physical obstacles. Thus several measurements are taken in for each position and each measurement contains the information of the RSSI levels for each access point, as is indicated in Figure 3. This process is repeated at different positions so the calibration process has several points/fingerprints and multiple measurements for each fingerprint.

The fingerprint information is represented by fi and is defined as follows:

(1) $$f_{i}=\{v_{i1},...,v_{ip}\}$$

where v*_ij_* is measurement *j* in fingerprint *i* from the total number of *q* measurements. It is defined according to Equation (2):

(2) $$v_{ij}=\{(m_{1},r_{ij1}),...,(m_{q},r_{\text{ijq}})\}$$

where m*_k_* is the detected MAC *k*, and *r_ijq_* is the RSSI level.

Using the information gathered from fingerprint, the system is able to scan the WiFi networks and locate the device in a given location performing probabilistic calculations. In the first step, the RSSI outliers of each MAC are removed in each fingerprint. Thus, a new fingerprint is constructed as follows:

(3) $$v_{ij}^{\prime }=\{(m_{c},r_{ijc}),...,(m_{v},r_{ijv})/Q_{1}-3*IQR<r_{\text{ijk}}<Q_{3}+3*\text{IQR}?\forall_{\text{ijk}}\}$$

where *Q*_1_ is quartile 1, *Q*_3_ is quartile 3, *IRQ* = *Q*_3_ − *Q*_1_.

The values of the RSSI levels are standardized for each MAC/BSSID in a fingerprint using Equation (4):

(4) $$r_{\text{ijs}}^{t}=\frac{r_{\text{ijs}}-{\bar{r}}_{i•s}}{S_{r_{i•s}}/\sqrt{n}}$$

where ${\bar{r}}_{ij•}=\frac{1}{q}\sum_{j}r_{\text{ijs}}$, $S_{r_{i•j}}=\sqrt{\frac{1}{n}{\left(r_{i•s}-{\bar{r}}_{\text{ijs}}\right)}^{2}}$, *q* is the number of measurements in fingerprint *i*.

In this way, each measurement is defined according to Equation (5):

(6) $$v_{ij}^{t}=\{(m_{c},r_{\text{ijc}}^{t}),...,(m_{v},r_{\text{ijv}}^{t})\}$$

During the location process the system has the measurement:

(6) $$v=\{(m_{c},r_{c}),...,(m_{v},r_{v})\}$$

The standardized measurement is generated in the same way as in Equation (4). It is applied for each BSSID *s* for each fingerprint *i*:

(7) $$r_{i,s}^{t}=\frac{r_{s}-{\bar{r}}_{i•s}}{S_{r_{i•s}}/\sqrt{n}}$$

Then, the value of α*_i,S_* is calculated; it represents the significance level to the fingerprint *i* and BSSID *s*:

(8) $$t_{\alpha_{is},n-1}=r_{i,s}^{t}$$

where *n* − 1 represents the freedom degree. If *n* > 30 then a normal distribution can be applied to calculate the probability. The probability of *v* ∈*f_i_* is defined as *P_fi_* :

(9) $$P_{fi}=\frac{\underset{s}{\text{∏}}\alpha_{i,s}}{\underset{i}{\text{∑}}\underset{s}{\text{∏}}\alpha_{i,s}}$$

To establish the associated fingerprint with the measurement *v*, *v* ∈*f_i_* if *P_fi_* is established as the maximum value.

**Figure 4.**
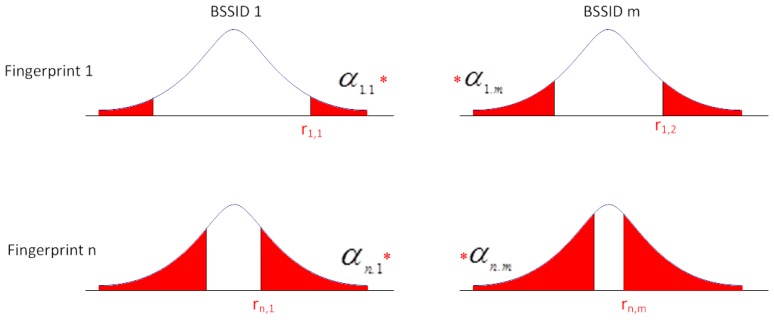
Process of calculating the likelihood of different fingerprints.

The process is graphically represented in Figure 4. Each row represents the probability of a fingerprint. For each row, the probability of being in the confidence interval of each BSSID in the fingerprint is shown.

A time series is established to keep transitional situations from making the system unstable. The system determines that a fingerprint *i* has been reached when the maximum value of the probability for fingerprint *i* is obtained for a predetermined number of consecutive times.

Although not mentioned above, the BSSIDs that are not detected in a given fingerprint are initialized with an RSSI value 20% lower than the minimum detectable value, thus the system differentiates between missing access points and detected access point with a low intensity.

Outdoors, the system uses GPS, which means that it would not be necessary to work with the WiFi to locate the user and determine the exercise the user is engaged in at that moment. Similarly, it is necessary to use accelerometers to determine if the user is actually walking or is traveling, for example, in public transport.

### 3.2. Physical Activity Monitoring System

The physical activity monitoring system will be used to determine when the user is in motion or otherwise stopped. It will also serve to stabilize the position of the user and prevent false movements that can result from changes in the WiFi signal. Mobile accelerometers are used to analyze the movement of the user and detect steps. There are currently many systems that detect and count steps, including Fitbit, Nike Fuelband and even mobile applications; however, they are unreliable because they erroneously record movements other than walking as steps. For this reason the use WiFi is necessary to avoid this problem, allowing for the use of accelerometers to determine whether the user is moving and to locate the user through the WiFi. Step Detectors are based on the analysis of the values obtained by the accelerometers. These values are then used to calculate the force vector according to Equation (11). The use of the force vector to detect the steps avoids analyzing the status of the gyroscope because the values of the accelerometers are given according to the axis of the device and not with respect to the axis of the earth. Furthermore, in order to facilitate the analysis, the value of the gravity acceleration is maintained:

(10) $$\vec{f}(x,y,z)=(x,y,z)$$

(11) $$\left|\vec{f}(x,y,z)\right|=\sqrt{x^{2}+y^{2}+z^{2}}$$

**Figure 5.**
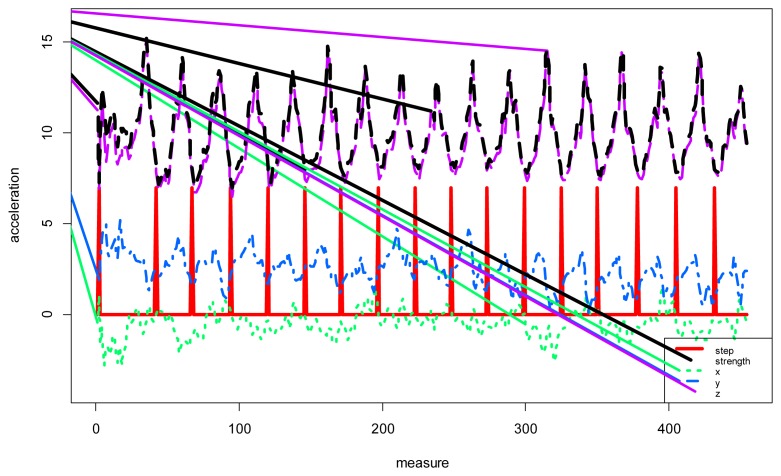
Variation of the accelerometers, force vector calculation and detection steps.

Figure 5 shows the value of the accelerometers in the *x*, *y* and *z* axes. The figure shows the force vector information in black, while the red line represents the detected steps. The values were obtained from walking with the mobile in hand. In the results section the system was analyzed with the mobile worn in the pocket. The detection process is simple: an upper and lower threshold is marked; then, when the value of the force vector exceeds first the upper threshold and then the bottom threshold, one step is counted. The threshold values indicate the sensitivity of the system. Figure 5 shows an example of step detection.

### 3.3. ECG System

**Figure 6.**
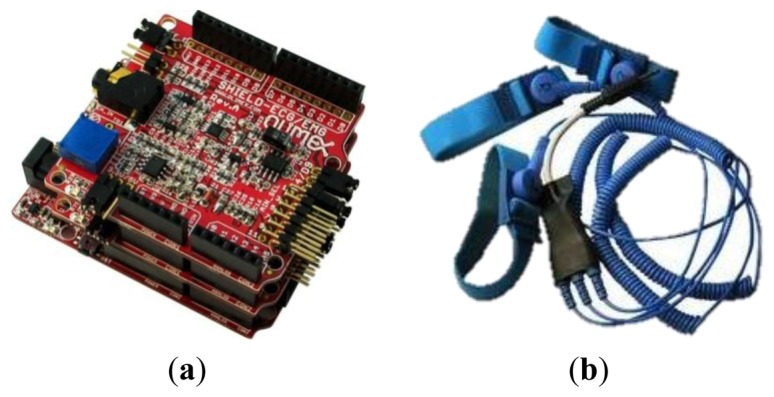
(**a**) Arduino, (**b**) electrodes.

The system comprises a Bluetooth Arduino Shield Arduino and Olimex SHIELD-ECG-EMG, as shown in Figure 6a. Furthermore, a few Electrodes SHIELD-ECG-EMG-PA are connected to the patient. The electrodes are shown in Figure 6b.

The Arduino firmware executes the source code of the ECG monitor role to read the data from the ECG. We used the Arduino programming language with Mstimer2 TimerOne and the libraries provided by the manufacturer. The firmware is used to obtain the values of the attached sensors in the shield and provides the information through a bluetooth connection.

**Figure 7.**
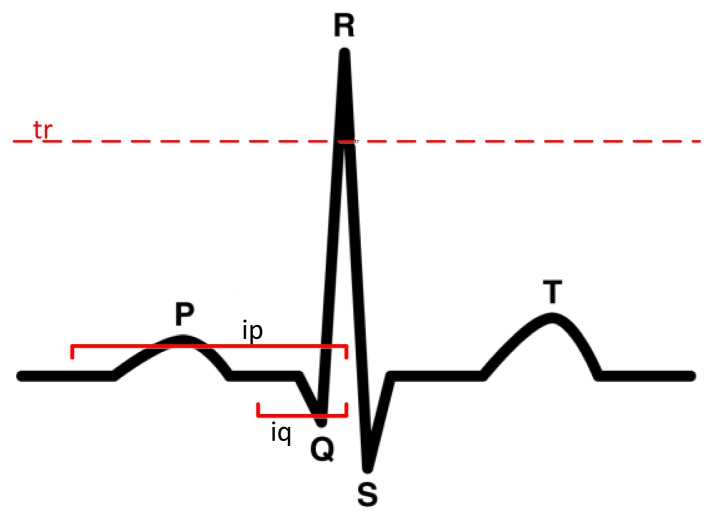
Waves P,T and complex QRS.

It is important to note that the hardware does not provide values in the same way as a professional ECG because it contains a lot of noise, which makes the detection of the P wave, the QRS complex and the T wave quite difficult, see Figure 7. The purpose of this module is more to monitor patients than to detect pathologies. The goal is have a low cost holter that can gather all patient information within a period of 24 h, and to then fuse this information with the values collected from the location system. This information will be useful to the doctor to discuss the patient's health status without requiring the patient to provide a detailed account of their behavior throughout the day. In any case, a basic functionality for detecting problems is included, although it is not necessarily a diagnostic system because the hardware is not considered safe for application in serious diseases. Previous studies [41] generated classifiers, such as SVM, from a trained database [46], and were able to reach an accuracy rate of near to 90%.

There are numerous studies to detect the QRS complex, as can be seen in [2,36–39]. The work done in [38] indicates that the location of the QRS complex is not an easy task due to noise and amplitude of the T wave, which may be confused in some cases with the QRS complex. Detection methods usually require dynamic management thresholds for detection of the complex when the signal is being analyzed in the time domain [36,38]. Other studies analyze the signal in the frequency domain [39], but basically all systems first filter the signal and then extract the complex QRS. The present study uses algorithms that allow extracting information in real time such as in [36,47]. The study done in [47] produced an error of 1.7% with the MIT -BIH Arrhythmia database; however, the algorithm proposed in [36] has a lower error in the different tests performed on the same database and was, consequently, selected to detect the QRS complex. Basically, the system includes four steps: (1) wavelet-based denoising with discrete wavelet transform (DWT) [40]; (2) linear highpass filtering (HPF); (3) non-linear lowpass filtering (LPF) to increase the QRS complex; and (4) decision-making based on the definition of a tr threshold according to the previous values. The threshold is used to generate decision rules to detect the complex QRS. The value of tr is defined according to the work in [36]:

(12) tr=α⋅γ⋅peak+(1−α)⋅tr

where α and γ are constant, and peak is the maximum local value.

As we can see, the process marked by Equation (12) does not require a lot of processing, and the highest computation time involves the application of the filters, although the process can be carried out in real time. The R value can be used to calculate Q and S by setting the search intervals the same way as in [36]. The process of finding the P and T waves is implemented according to [48], and consists of defining intervals around R. Other algorithms use classifiers such as SVM [49] to locate these waves; however, its use is discarded to avoid using black boxes in the analysis and to be able to perform the processing in real-time.

To detect anomalies, some basic rules were introduced based on the type of problem detected. These rules could be automatically generated from a case base using a J48, for example, but we prefer to rely on expert knowledge instead of an automatic generation to avoid incorrect detections associated to the database. We specifically analyzed the following: sinus tachycardia up to 150 beats, sinus arrhythmia beats (variations according to the breathing apparatus were easily observed), SA nodal blocking a pause in multiple pp intervals (sinus pause is not a pause interval, it is a multiple of *p*). When an abnormality is detected, the agent in the mobile phone sends an alert to the multi-agent platform.

The information managed information in each measurement *e_i_* to generate the rules is shown in Table 1.

For example, a sinus tachycardia (up to 150 beats) is triggered if the following inequality holds:

(13) $$n60.000/\sum_{i=k}^{k+n}R_{i}>150$$

*k* would be the initial element of the time series, and n the number of factors used to calculate the time series.

For SA nodal blocking a pause in a multiple of p-p interval, the rule would be defined as follows:

(14) $$\frac{P_{i}}{P_{i-1}}>1.5\&\&P_{i}\%P_{i-1}<e$$

where % represents the remainder of dividing *P_i_* and *P_i_*_−1_ and *e* represents the margin to determine whether the value is a multiple of two consecutives p waves.

The same process would be followed with other pathologies. All rules are defined in advance but, as mentioned above, they could be generated automatically with decision rules, a SVM, Bayesian networks or any other classifier using a database with enough information about all the pathologies.

### 3.4. Monitoring System with Television

Nowadays most elderly people require constant attention and care, but do not have a caregiver who can permanently reside with them all day. Continuous advances in hardware design and development in recent years have caused a decrease in the manufacturing costs of sensors and other materials that can be used by researchers to design assistance systems especially oriented to the elderly population, even providing remote care services. Our system introduces a new television-based interactive model to interact with elderly people. We present an innovative solution based on a remote control device for TV that incorporates a reduced number of buttons. Interaction through television was chosen in this study because users are typically familiar with all the operations, and it is a device that can be found in most homes.

**Figure 8.**
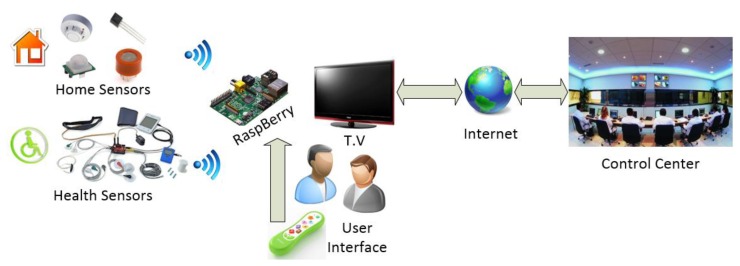
Interaction system based on television.

The main advantage of the proposed system compared to the existing ones in the market such as GoogleTV or AppleTV is the capacity for personalizing the services that can be offered, as well as the low cost of the proposed solution. Nowadays, existing systems focus on specialized multimedia services, renting movies, buying video games, but it is not possible to find and open, low-cost care system for the elderly people. Below, Figure 8 shows the different components of the proposed solution:

The proposed solution incorporates a low-cost computer (30$), which facilitates robustness and scalability. We propose an architecture that can be accepted and integrated with existing systems by the vast majority of people interested in home care solutions in their homes. The Raspberry mini-computer is the physical support for the architecture. One of the main advantages of the proposed architecture is its ability to embed agents in the Raspberry, enabling distributed communication and the possibility to connect remotely with the patient's home, thus obtaining context-aware information very easily. In addition, the agents will be able to obtain real-time context-aware information coming from different sensors that are connected to the Raspberry wirelessly such as gas, temperature, fire, presence sensors, or sensors which are geared to the caregiver, such as, oxygen meter, blood pressure, ECG, accelerometer … *etc*. In this case study the system incorporates accelerometers and EKG. The use of the Raspberry device allows us to easily instantiate embedded agents and connect them with the PANGEA multi-agent architecture. In our case we have embedded two agents, one of them designed to analyze data obtained from an EKG device and the TV. We have chosen Raspberry as the algorithm designed to interpret the data requires a high computational cost and the use on an Arduino device is not recommended in this case; and another agent that interacts with the elderly by means of the television. The Raspberry is also connected to Internet and can be connected to any existing TV using an HDMI cable.

The use of an agent that interacts with the user via TV allows us to issue warnings or notifications whenever necessary. For example, remind the user to take the medication, or ask if any assistance is needed when an anomalous situation is detected.

One of the advantages presented by the proposed architecture is that the information extraction algorithms that monitor the various sensors installed in the home of the elderly patient can be executed without computational restrictions. And in case of anomaly detections, a notification or alert can be automatically sent to the emergency control center. In this case, the agent with the EKG analysis role sends alerts to the control centers when it detects an anomalous situation. The doctor may send a message to the users in order to initiate interaction. Additionally, the system may provide the user with reminders, for example, to use the holter.

**Figure 9.**
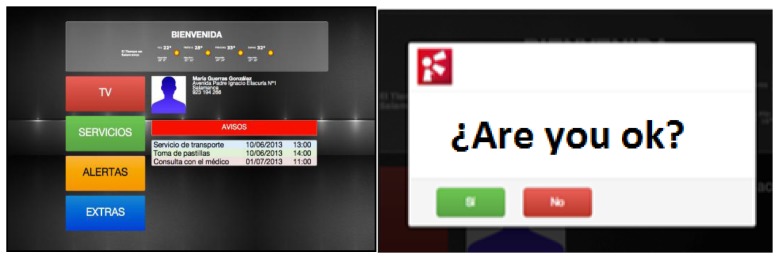
Interaction with the user through television.

Figure 9 shows a screenshot of the proposed system working in the case study, where a warning message is sent to the user. This message is received by the agent with the TV role and is displayed on the TV. Even if the user is watching a movie at the moment of receiving the notification, the information is superimposed on the TV programme and made visible to the user.

The interface agent uses a web page based on HTML5 which makes the interface easily customizable remotely, allowing the dynamic personalization of the 4 buttons to interact with the system.

The use of devices that allow embedding agents with a low cost to develop services for people, opens up a vast array of possibilities, allowing monitoring for the elderly without the high cost of entry into a residence or an assistant is present all day.

## 4. Case Study

The system was tested on a person residing in an 85 m^2^ flat. The necessary hardware for the tracking system, the motion detection system, ECG system, and TV system were all deployed. Each hardware element and its corresponding price are indicated in Table 2.

**Figure 10.**
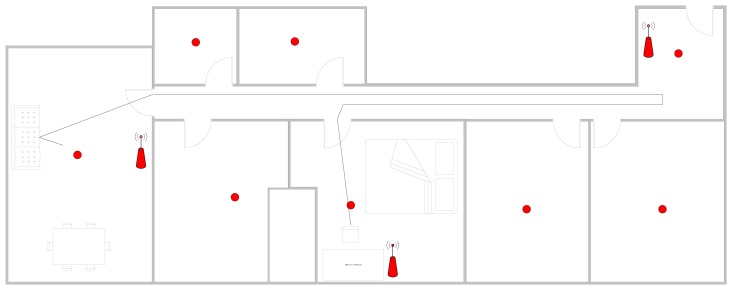
Path followed in the home.

TL-WR740N routers were specifically selected because they can be flashed with dd-wrt, which makes it possible to modify dBm. Dd-wrt can reduce the dBm in higher accuracy is required. Each of the routers is distributed so that the maximum possible area is covered. Figure 10 includes the floor plans for the flat in which the tests were carried out. The antennas represent the routers. As we can see, the routers are located at both ends of the floor and in the middle.

The electrocardiogram has three electrodes: two placed on the user's wrists and the third one on an ankle. The electrodes in the current version are connected by wires to the Arduino, but wireless communication is also available for a much higher price. The Arduino device connects with a mobile via bluetooth; however, the user must carry the mobile device for the location and movement system to function properly and send the information from the ECG.

The ECG has not been tested on a patient with heart disease because the system is still in its testing phase. Nevertheless, the results obtained from the ECG have been validated by doctors who also consider that the use of the system would be feasible in a diagnostic application. The results were satisfactory and indicated that the *p*, *t*, and complex QRS waves could be used, although the noise is higher than a professional holter system.

## 5. Results and Conclusions

The system has been implemented according to the case study described in Section 4. First, we validated each of the components and then the entire system. Individual validations were performed to ensure that each component worked correctly and the results were satisfactory. The same process was applied to validate the location system, the physical activity monitoring system, the electro cardiogram, and the proper functioning of the television system.

The first system tested was the location tracking system. The first step was to calibrate the system, which consisted of taking measurements at the points indicated in picture 3. Each point corresponds to a fingerprint, and for each fingerprint 50 measurements were taken. Measurements were taken by opening and closing the door of the room in which the measurements were taken. Additionally, one of the access points was placed in the hallway to avoid signal changes associated with the opening and closing of the doors. The other doors were half open to make measurements. A LG Nexus 4 was used during the calibration process to obtain the RSSI signals. This device can scan the WiFi signal faster due to the hardware and the fact that it is an android version. The measures can be used with other devices with high precision; in this case we have used an LG Nexus in the calibration process and an HTC Sensation XL during the test. The router TP-LINK TL-WR740N was flashed with DD-WRT in order to modify transmission power to 6 dbm and provide better accuracy; the original value was 20 dbm. There is an additional router to connect the mobile phone to the WiFi; this router is not used in the location system although it could be incorporated to improve the accuracy.

The tracking system was compared with classifiers based on Bayesian networks [7], SVM [50]. The application of the time series was omitted as we considered it irrelevant for this test. These classifiers were selected because they had been used in other studies on location systems. To make a more realistic validation, a 5 × 2 cross-validation was not performed as in previous studies [51]. Instead, new measures were taken as for a new calibration. We took 50 measurements per fingerprint, each of which was classified with the constructed classifier in the first data set. The reason for not taking both sets and applying a 5 × 2 cross validation was to estimate measurements corresponding to different times. This allowed us to change the environment by opening and closing doors, modifying or moving furniture, and creating new estimates with these set of data. This process was repeated 5 times, which allowed us to statistically validate differences between systems. The differences were analyzed by Mann-Whitney. Table 3 shows the results obtained for each of the sets of validation created. The comparison between pairs of methods can be seen in Table 4. Bold values indicate that the differences are considered significant.

The results are similar to those obtained from the Bayesian network; however, the results obtained by SVM are significantly worse. The Bayesian network must previously apply a discretization of the values and the results depend in large part on the discretization process. The validation of the GPS tracking system lacks scientific interest and is therefore not included in the paper.

**Figure 11.**
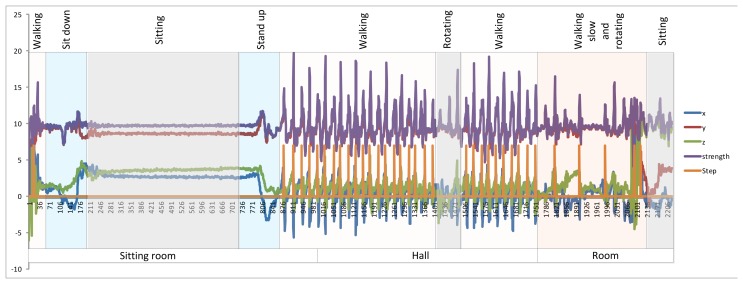
Detection steps based on different actions.

To validate the physical monitoring system we proceeded to perform sequences with basic daily activities to determine whether the system was able to detect movements associated with steps. The upper and lower thresholds were fixed to 10.3 m/s^2^ and 9.1 m/s^2^ respectively. Figure 10 traces the user's movements, while Figure 11 shows the sequence of actions and the performance of the step detector, in which the user moves from one room to another walking down the hallway. The user began in a standing position, walked to the sofa and sat down. The user then stood up, left the room, walked to the end of the hall, turned back and entered one of the rooms. Inside the room, the user walked slowly to the back and sat on the chair. The mobile was located in the user's pocket with no particular concern as to its position. As seen in the figure, the steps marked in orange were easily detected according to the movement of the user, the frequency indicates the speed.

As we can see, the physical monitoring system works quite well and is able to determine the movement with high accuracy. The user is located through WiFi, so the tracking system stops updating the position after 3 s of not detecting movements; however, it is easy for the system to erroneously count steps when the user is not actually walking. For example, steps are erroneously detected when the user is preparing dinner and bends over to pick something up. Over the course of one hour, the system detects 573 steps. The number of steps detected correctly was 527, while 21 steps were lost.

**Figure 12.**
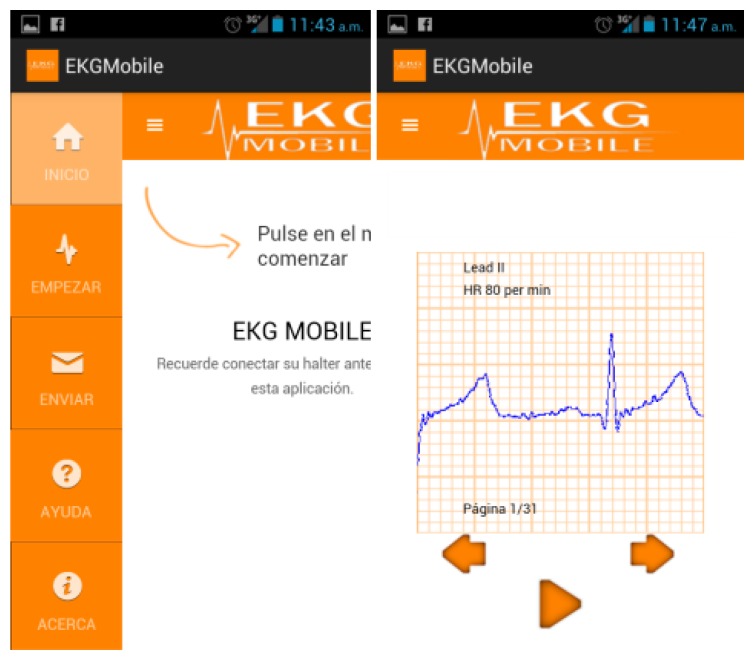
ECG mobile application.

Figure 12 shows the ECG at runtime. The user can monitor the values in real-time and they are interpretable as a brief training received from the staff. The ECG shows the P wave, the QRS complex, and the T waves.

The ECG system sends data to a central location where a doctor can monitor a set of patients. The element that allows connecting the health center to the patient's home is Raspberry *Pi*. When a rule is triggered, the system sends an alert to the mobile phone and to the television. Figure 9 shows an alert of the system in the TV when a rule has been triggered.

**Figure 13.**
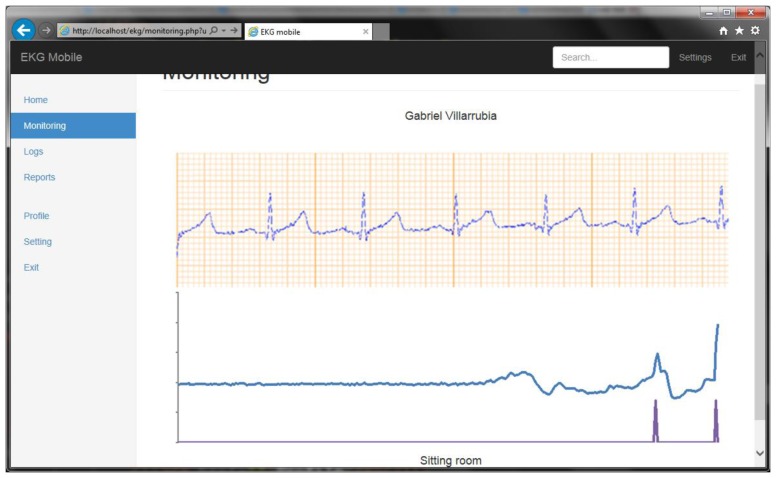
Remote monitoring of the user status.

The doctor can visualize and monitor the status of the patient. When an anomaly is detected, the system sends data automatically, although the user may also send the data. Figure 13 shows the information of the ECG sent to the doctor, and includes information about the user's movements.

The system can monitor user behavior. The monitoring system functions include locating the user, monitoring physical activity, electrocardiogram; it is also able to interact with the user through the television. All hardware used is low cost, making at-home installation easily affordable. Moreover, the setup simply requires taking some measurements during 1 min in each of the rooms. The probabilistic calculation of belonging to a different fingerprint allows better performance with regard to changes of the signal level as compared to other classifiers such as decision trees. This is due to the fact that the probability is distributed between different fingerprints. Modelling the systems by means of probability allows the use of different mobile devices (with different levels of signal receiving), thus preserving the accuracy of the location system. This accuracy remains more or less constant because the probability does not change (since it would be distributed in a similar way). However, in alternative potential solutions, such as decision trees, this issue can become problematic because the signal levels play an important role in the rules that determine the final location.

The tracking system and monitoring systems provide information with enough detail to determine the movement of the users. Alternatively the system could use the FitBit or another similar bracelet.

The ECG provides enough results to analyze the electro, although it produces some noise. It would be possible to use better hardware, although it would no longer be a low-cost product, which is why this alternative was rejected. Our future research will focus on improving the detection of arrhythmias systems. We will also analyze different possibilities of incorporating a new mechanism to automatically generate rules and knowledge. The current version of MIT-BIH Arrhythmia as focused on incorporating a large amount of information and functionalities that are of interest to the medical staff. The next step is to make advancements in the intelligence of the system, incorporating an expert system that can help to define rules and knowledge in order to improve the training process and to avoid potential problems with erroneous values in the database.

Finally, the Pangea architecture makes it possible to interconnect the elements of the platform very easily because it can incorporate agents in lightweight devices such as Arduinos and mobile phones. Our future work will focus on improving the automatic processing of the ECG system, although it should not be used as a diagnostic mechanism because the hardware has not been approved.

## Figures and Tables

**Table 1.** EKG waves and times.

**Wave**	**Time**
*P_i_*	Milliseconds from the last value *P_i−1_*
*Q_i_*	Milliseconds from the last value *Q*_i−1_
*R_i_*	Milliseconds from the last value *R_i_*_−1_
*S_i_*	Milliseconds from the last value *S_i_*_−1_
*T_i_*	Milliseconds from the last value *T_i_*_−1_
*Dpq_i_*	Milliseconds between the maximum value of the *P* wave and minimum value of *Q* wave.
*Dqr_i_*	Milliseconds between the minimum value of the *Q* wave and maximum value of *R* wave.
*Drs_i_*	Milliseconds between the maximum value of the *R* wave and minimum value of *S* wave.
*Dst_i_*	Milliseconds between the minimum value of the *S* wave and maximum value of *T* wave.

**Table 2.** Hardware.

**Hardware**	**Quantity**	**Unit Price**
Router TP-LINK TL-WR740N. For the location system and to connect the various elements to the data network, either through WiFi or cable.	3	15€
Any Android telephone with WiFi, GPS and an accelerometer would suffice. LG Nexus 4.	1	200€
Arduino Bluetooth	1	40€
Olimex Arduino Shield SHIELD-ECG-EMG	1	20€
Electrodes SHIELD-ECG-EMG-PA	1	8€
Raspberry PI	1	40€

**Table 3.** Colum *ai* represents the number of elements correctly classified of *ai* training with *bi* and column *bi* the number of elements correctly classified of *bi* training with *ai*, *i* is the repetition.

**Method**	***a1***	***b1***	***a2***	***b2***	***a3***	***b3***	***a4***	***b4***	***a5***	***b5***	**average**
**Proposal**	386	385	378	373	387	376	379	386	379	382	381.1
**Bayesian**	382	378	369	374	378	379	381	375	376	368	376
**SVM**	353	348	357	339	368	359	359	349	356	352	354

**Table 4.** Mann-Whitney test.

**Method**	**Proposal**	**Bayesian**	**SVM**
**Proposal**	1	**0.04426767**	**0.00017962**
**Bayesian**	**0.04426767**	1	**0.00020871**
**SVM**	**0.00017962**	**0.00020871**	1
